# The public goods hypothesis for the evolution of life on Earth

**DOI:** 10.1186/1745-6150-6-41

**Published:** 2011-08-23

**Authors:** James O McInerney, Davide Pisani, Eric Bapteste, Mary J O'Connell

**Affiliations:** 1 Molecular Evolution and Bioinformatics Unit, Department of Biology, National University of Ireland Maynooth, Co. Kildare, Ireland; 2 Uniteé Mixte de Recherche, Centre National de la Recherche Scientifique 7138, Systeématique, Adaptation, Evolution, Universiteé Pierre et Marie Curie, 75005 Paris, France; 3 Bioinformatics and Molecular Evolution Group, School of Biotechnology, Dublin City University, Glasnevin, Dublin 9, Ireland

## Abstract

It is becoming increasingly difficult to reconcile the observed extent of horizontal gene transfers with the central metaphor of a great tree uniting all evolving entities on the planet. In this manuscript we describe the Public Goods Hypothesis and show that it is appropriate in order to describe biological evolution on the planet. According to this hypothesis, nucleotide sequences (genes, promoters, exons, etc.) are simply seen as goods, passed from organism to organism through both vertical and horizontal transfer. Public goods sequences are defined by having the properties of being largely non-excludable (no organism can be effectively prevented from accessing these sequences) and non-rival (while such a sequence is being used by one organism it is also available for use by another organism). The universal nature of genetic systems ensures that such non-excludable sequences exist and non-excludability explains why we see a myriad of genes in different combinations in sequenced genomes. There are three features of the public goods hypothesis. Firstly, segments of DNA are seen as public goods, available for all organisms to integrate into their genomes. Secondly, we expect the evolution of mechanisms for DNA sharing and of defense mechanisms against DNA intrusion in genomes. Thirdly, we expect that we do not see a *global *tree-like pattern. Instead, we *expect *local tree-like patterns to emerge from the combination of a commonage of genes and vertical inheritance of genomes by cell division. Indeed, while genes are theoretically public goods, in reality, some genes are excludable, particularly, though not only, when they have variant genetic codes or behave as coalition or club goods, available for all organisms of a coalition to integrate into their genomes, and non-rival within the club. We view the Tree of Life hypothesis as a regionalized instance of the Public Goods hypothesis, just like classical mechanics and euclidean geometry are seen as regionalized instances of quantum mechanics and Riemannian geometry respectively. We argue for this change using an axiomatic approach that shows that the Public Goods hypothesis is a better accommodation of the observed data than the Tree of Life hypothesis.

## Background

The "Tree of Life" hypothesis has been in existence for most of the last two centuries and is one of several hypotheses that have been put forward to explain the diversity of life on the planet [1]. In its recent practice, this theory focusses on the vertical inheritance of genes from parent to offspring and the continuous division of lineages in the process of speciation. The theory was largely formulated in the days when the science of systematics was mostly concerned with the analysis of plant, fungi and animals and the study of evolution was essentially focussed on the study of these three eukaryotic lineages. Indeed, arguably the greatest contributor to the definition of the species concept, Ernst Mayr, was so adamant in pinpointing that his views specifically applied to sexual organisms that he clearly titled his major work on speciation mechanisms "*Systematics and the origin of species from the viewpoint of a Zoologist*" [2].

Microbiologists engaged in a long and largely unsatisfying search for the tree of prokaryotic life for most of the 20th Century [3,4] (also see [5] for review). Woese detailed this search in his treatise on bacterial evolution [6], where he also wrote about the ideas and false-starts that arose from time to time in the earlier part of the century. The 1970s had resulted in significant developments in the sequencing of genes and this led Woese and others to the belief that there was a new tool available to systematists that would ultimately lead to a satisfactory and detailed resolution of the entire Tree of Life [7-9]. The fine-grained picture of the tree of life was rapidly being brought into focus by the use of ribosomal RNA sequencing and analysis. Indeed, so powerful was this line of argument that today, the world's most targeted gene for sequencing is the small subunit ribosomal RNA gene. However, and remarkably so, the most sequenced and most ubiquitous kind of gene in the world is not the small subunit ribosomal RNA gene. Transposases, typically known to function by frequent movement from one genetic element to another are the most abundantly discovered genes in metagenomic studies where sampling of genes is undirected [10]. This discrepancy between the most abundant marker sequenced in the traditional phylogenetic framework and the most abundant genes actually obtained in nature by chance, suggests that the scope of phylogenetic analysis is focussed on the analysis of certain genes, while if a different perspective was taken - to focus on the most abundant genes - then a different interpretation of evolutionary history might be more readily obtained.

Commonly, by the 1990s, the optimism surrounding the reconstruction of a tree of life was giving way to a more realistic picture of life on the planet. Hilario and Gogarten [11] and Martin *et al. *[12] pointed out remarkable inconsistencies in molecular phylogenies derived from ATPase and Glyceraldehyde-3-phosphate dehydrogenase genes when these phylogenies were compared with ribosomal RNA phylogenies. A genome analysis showed that a substantial portion of the known *E. coli *genome was acquired by horizontal gene transfer since its separation from *Salmonella *[13]. At the end of the century, Doolittle [14,15] and Martin [16] summarized these growing problems with the tree of life model. Soon afterwards, serious efforts were being made to identify interspecies gene transfer events and to quantify the extent of HGT in prokaryotic genomes [17-19] and phylogenetic tree diagrams have been increasingly giving way to network models of genome evolution in prokaryotes [20,21]. It must be pointed out, however, that the focus on HGT events has been strongly criticized [22,23] and some congruence between gene trees is easily found [24].

It is not the case, however, that network diagrams are being universally employed and indeed they still appear in only a minority of studies that deal with the molecular systematics of prokaryotes. Tree diagrams, inferred from a subset of genes with a wide or universal distribution are still the most commonly used models for prokaryotic evolution [25], though it has been pointed out that usually these diagrams are only constructed from less than one percent of the genome of these organisms [26]. When a larger portion of the genome is used, then the resulting tree diagram is highly dependent on the method used in its construction [27] and in any case, no strongly-supported nodes are found when moving towards the base of this tree [24,27]. In the middle ground are studies that try to reconstruct a tree or forest of trees in the presence of HGT events [28-33].

What is becoming increasingly obvious is the need to either improve the Tree of Life model, if that is indeed possible, or replace it with one (or several) hypotheses that better fit the data. At the moment the interpretations of this model seem to be straddling the middle ground - there is a great Tree of Life (*sensu *Darwin, Lamarck, etc.) but it has annotations and complications superimposed on its great frame, caused by interspecies gene transfer. The problem with this model is that it is becoming increasingly implausible. Recent estimates show, for instance, that *Escherichia coli *as a species uses approximately 18,000 genes [34,35], while the percent of gene families that are now known to be found in every *E. coli *is just 6% of the total (see also Beauregard-Racine *et al. *[36]), though a typical *E. coli *strain only possesses 4,000-5,500 genes. This kind of scenario is seen again and again during genome resequencing projects where multiple strains of the same species are sequenced. A minority of the genes that are found in a prokaryotic species are found in just one genome of that species. As a consequence, the Tree of Life hypothesis has been modified extensively from its original description, in order to avoid its rejection. We now know - unlike the originators of the hypothesis almost two centuries ago - that the main process of genome innovation for many of the evolving entities on this planet is not vertical descent, rather it is recombination and gene acquisition [37]. Genes and genomes did not form part of the original formulation of the Tree of Life hypothesis and processes such as horizontal gene transfer and mobile genetic elements such as viruses, plasmids and transposons obviously did not feature. In order to accommodate these newly discovered, important features, the hypothesis has been stretched to fit the data, however, given our knowledge of the data, it seems that the elastic limit of the original hypothesis has been passed.

In Darwin's formulation of the Tree of Life hypothesis, he said that he attempted "[...] *to show that there is a constant tendency in the forms that are increasing in number and diverging in character, to supplant and exterminate the less divergent, the less improved, and preceding forms*." This particular quotation gets to the heart of tree-thinking: that evolving entities would always diverge away from one another and that evolution is a *process *of divergence. To put it another way, all formulations of the Tree of Life hypothesis have at their core the basic tenet that the pattern of diversity that we see on the planet is caused by a tree-like evolutionary process and the differences in these formulations is to be found in how much they allow deviation from this central idea. Almost without exception, efforts to describe the diversity of life on the planet have focussed on the construction of this tree. The tree has been portrayed as a strictly bifurcating tree, as a fuzzy tree, as a tree with cobwebs hanging from it and so forth. Likewise, the number and kinds of evolving entities has changed over time, prokaryotes being largely ignored initially (see [5]), mainly due to the difficulties of generating interpretable trees from the available data.

The current attempts at constructing the tree of life can be roughly divided into four approaches (though, other classifications of the approaches are easily constructed). Firstly, there is the tree as exemplified by the small subunit ribosomal RNA gene [6]. Next, there is the multi-gene approach using widely distributed genes, usually of informational function [25]; the third approach is to search for the biggest observable trend embedded in the data [31], and the fourth is to construct phylogenetic supertrees, the so-called tree-from-trees method. These approaches have different meanings and care must be taken to interpret what they say. When the ribosomal RNA approach was first advocated by Woese and co-workers, the ubiquity of the gene and its attractive properties in terms of rapidly and slowly-evolving sites, its conserved structure and its supposed recalcitrance to horizontal gene transfer meant that it was simply being used as a surrogate for the evolution of the entire organism, a position that is no longer tenable. Using multiple genes in a concatenated superalignment is designed to overcome the limitations of using a single gene, however the interpretation of this tree is somewhat similar to the interpretation of the rRNA tree and this approach has been criticised as a "Tree of 1%", not a tree of life [26]. The Statistical Tree of Life (STOL) and phylogenetic supertrees are constructed from a much larger sample of genomes and attempt to either construct a single tree (supertree approaches) or a statistical trend that is tree-like from a large sample of a genome. The interpretation of these structures is somewhat difficult, though they may approximate a "Tree of Cells". Unfortunately, it is now necessary to carefully read each manuscript to find out what the authors are calling the "Tree of Life".

### Presentation of the Hypothesis

Clearly, if it is our ambition to question or test the Tree of Life hypothesis, then many approaches are inappropriate because they conflate the explanandum and the explanans (as already pointed out by Bapteste and Doolittle [38]) - constructing a tree, however slender, weak or fuzzy and using this tree to provide proof that there is a tree. However, with few exceptions [21,39], all approaches to date suffer from starting at the same place - assuming that there is a fundamental tree-like structure at the heart of biological evolution and then invoking *ad hoc *criteria to explain data that does not conform (the presence of plasmids, viruses, horizontal gene transfer, genome fusion, endosymbioses and so forth). Tree of Life efforts have generally had no regard for mobile genetic elements such as plasmids and viruses and their classification and evolution is typically discussed in a completely separate body of literature (e.g. [40]).

Tree-like patterns undeniably exist but a tree is the incorrect starting proposition and no matter how much we customize the interpretation of this metaphor, we will not be able to make it fit the observed data. In this manuscript, inspired by patterns observed in genomic data, we present a model of evolution explaining both the reason why genomes have an almost endless combination of genes and why modest tree-like patterns are seen when some small subsets of the data are compared with each other. First of all we present two axioms (uncontroversial starting points that are self-evidently true) on which our model is based.

#### Axiom 1

All evolving entities should be included in a model that aims at providing the highest-level evolutionary picture.

#### Axiom 2

Genes move both horizontally and vertically.

For axiom 1, we include all nucleotides that are part of a replicating system - chromosomal, plasmid, viral and so forth. Given that these two conditions are uncontroversial, any hypothesis or model that purports to describe biological evolution on the planet should be compatible with these two desiderata. With these axioms in mind, we move on to describing the public goods hypothesis, which is our replacement for the Tree of Life hypothesis.

### The Public Goods model of evolution

While John Locke wrote about ownership of property and its governance in 1690 [41], it is usually Paul Anthony Samuelson, the first American to win the Nobel Prize in Economics who is credited with being the person who introduced the notion of public goods and private goods [42]. In drawing the distinction between different kinds of goods, Samuelson said:

"[...] I explicitly assume two categories of goods: ordinary private consumption goods [...] which can be parcelled out among different individuals [...] and collective consumption goods [...] which all enjoy in common in the sense that each individual's consumption of such a good leads to no subtraction from any other individual's consumption of that good [...]."

Today four categories of goods have been characterized (see table 1). These goods differ in two of the features that can be said to describe goods: whether the good is rival and/or whether the good is excludable. Rival refers to the availability of the good. A good is non-rival if the consumption of the good by one individual does not reduce the availability of that good for another individual. A good is non-excludable if it is impossible or at least very difficult to exclude the good from being available to everybody. The air we breathe is a good example of a public good. The consumption of clean air by one individual does not greatly reduce the availability of air for other individuals, so this makes air a non-rival good and it is impossible to effectively exclude all other individuals from accessing air, so this makes air a non-excludable good. Consequently, we think of air as a public good.

**Table 1:** The four categories of goods classified according to the criteria of whether they are rival and/or excludable

	*Excludable*	*Non-excludable*
*Rival*	Private goods	Common goods
*Non-Rival*	Club goods	Public goods

In order to elaborate on our hypothesis, we must first of all explore genes to show that some may be considered as public goods. For the rest of this manuscript we will use the term "gene", but in reality we are talking about any sequence of nucleotides that can reasonably be considered to be a functioning unit or good (a gene, portion of gene, promoter sequence, etc.). Genes can be classified into homologous families and sub classified into orthologous and paralogous genes (also other classifications such as ohnologs and xenologs exist), but for the purposes of simplification we will speak generally about homologs, defined by Richard Owen as "the same organ in different animals under every variety of form and function" (Owen, 1843), though of course, we are speaking about genes and not animal organs. As far as we know, there is no limit to the number of gene copies that can exist for any given family of genes on Earth, though obviously we see that individual evolving entities have finite genome sizes. We see some gene families widely distributed (say, transposases, informational genes [25] or enzymes such as Rubisco [43]) and conversely, many genes have so far been only seen in a single sequenced genome (personal observation). In addition, a particular gene copy from a homologous gene family can be used without "using up" the gene family, in the same way that molecules of air can be used by some organisms without appreciably using-up the air. When gene copies are moved laterally, they are usually moved using a copy-and-paste mechanism (e.g. in plasmids, phage, class I transposons, outer membrane vesicles and gene transfer agents), not a 'stealing' mechanism such as cut-and-paste (e.g. class II Transposons, nanotubes [44]). These features seem to suggest that some genes are non-rival - the use of such a gene by one organism does not preclude its use by another organism.

If we furthermore consider the observations that have been made on the widespread and easy gene availability to single-celled prokaryotes: conjugation by plasmids [4,45], the transformation of prokaryotes by phages [46], the natural competence of some organisms like *Neisseria *[47], the secretion of outer membrane vesicles packaging DNA in bacteria [48,49], the ability of gene transfer agents to release small sections of DNA into the environment [50], particularly, it seems in the oceans [51], the sharing of DNA material through nanotubes [44] and the experimental demonstration that barriers to forced gene transfer by cloning are almost non-existent [52,53], these empirical observations all point to many genes having the property of being non-excludable. That is to say, it is very difficult for prokaryotes to completely prevent other prokaryotes from obtaining a particular gene. Indeed, the evolution of CRISPR elements [54] and restriction-modification systems [55], themselves laterally transferred [56] testifies to the evolutionary success of (and obvious need for) mechanisms that protect against the (passive or aggressive) acquisition of genes.

Genuine public goods (non-excludable and non-rival) are relatively rare, often somewhat intangible and sometimes context-dependent. Knowledge is considered to be a public good - it is difficult to prevent the spread of knowledge and facts don't get "used up" if many people know them. In an earlier example, we cited clean air as a public good. However, in the context of scuba divers under water, bottled air is not a public good, it is a private good. In another example - this time somewhat analogous to gene sharing - it is asserted that file-sharing on the internet has the hallmark of a public good (the files are spread by a copying mechanism and once a file is freely available on the internet, it is very difficult to completely prevent people from accessing that file and making copies available for further sharing) [57]. Legislation and other means could conceivably restrict file-sharing on the internet and therefore, while it might be considered to be a public good, its classification might be context dependent.

We may conclude, therefore, that if some genes are non-excludable and non-rival, they are public goods. Likewise, it can be argued (and empirically tested) that some genes might be *de facto *non-rival but excludable to some extent and in some contexts, therefore, they might be better described as club goods rather than public goods.

A protein-coding gene could be excludable, for instance, if it uses a genetic code that is unique to a particular group of organisms. This DNA sequence could produce a defective protein in an organism with a more orthodox (universal) genetic code and consequently the gene would not be fixed in that population, but would be lost. Excludability of the gene in this case would come from an intrinsic characteristic of the molecular sequence and would be somewhat independent of function. In another scenario, a protein might only function, say, in the absence of oxygen and therefore all aerobic organisms would be excluded from using the gene that encodes this protein. In this case, the function of the encoded gene is the feature that would make a gene into a club good. In a recent study it has been shown that the connectivity of a protein in a protein-protein interaction network plays a large part in whether it can be successfully transferred to another species [58]. It is possible that genes encoding very highly connected proteins are effectively excludable and therefore could be considered club goods.

We might also consider the case where the composition of the club plays a central role in whether a gene remains a public good or becomes a club good. Symbiotic association of multiple lineages, have frequently been observed and indeed, these organisms might be "addicted" to one another. For instance, phylogenetically heterogeneous communities (or coalitions [59]) found in gut microbiomes, comprised of archaebacteria and eubacteria, have converged in their repertoires of carbohydrate-active enzymes to adapt to shared challenges. This is in large part thanks to lateral gene transfer mediated by mobile elements [60]. Such genes whose exchanges are restricted between members of the coalition, but that are not so widely spread outside the coalition, constitute a particular type of club goods, because of the original nature of the club itself.

We can call these genes "coalition goods", because they might represent a class of genes that, although they are potentially public goods, are generally excluded from being used by cells outside of the coalition, which is commonly illustrated by metagenomic studies reporting stronger functional signatures in microbial communities over phylogenetic signatures, i.e. in which the presence of particular genes coding for a certain function is more essential than the presence of a particular lineage for the community to evolve in a sustainable way [61]. The dependency of the different lineages in the coalition on one another thriving under particular selective pressures may be sufficient to set up a barrier to gene availability. Therefore such coalition goods are ultimately bounded by the spatial distribution and the ecological interactions of the members of the coalition. The genes might be theoretically public goods, but practically, they are club or coalition goods.

Most importantly, the notion of a gene as a public good or a coalition good is entirely at odds with the Tree of Life hypothesis, which implies that genes are either private goods (excludable and rival) or club goods (excludable and non-rival) as long as we define the club to be a single clade (monophyletic group).

### How are the goods kept in public ownership?

To be sustainable, public goods need to last and remain available. In the case of genes, the maintenance of these public goods and coalition goods is taken on board by the organisms and mobile genetic elements. There is a single genetic code (allowing for the numerous variants, which are mostly, though not always, found in limited kinds of organisms - *Mycoplasma *and *Spiroplasma *for instance have a variant of the genetic code). This genetic code is maintained by almost all the organisms that use it. Mutations to genes that introduce stop codons are generally weeded out by natural selection. Genes that have proved useful to organisms, mobile elements, or their coalitions have been replicated, while acquisition of deleterious genes has led to the extinction of their carriers. Therefore, organisms and mobile genetic elements patrol the public goods and these goods are maintained over some evolutionary time by natural selection. Nonetheless, a turnover in the composition of the gene families is expected, because the nature of selective advantages can evolve over time, i.e. after oxygen level raised in the atmosphere, numerous gene families involved in O_2 _metabolism likely became public goods [62]. The same logic applies for antibiotic resistance genes, etc. This is another feature of genes that legitimise the classification of some genes as public goods, though it is not a necessary feature. If genes were private goods, then we might expect alternative genetic codes to evolve over time thereby helping to keep the private goods private. This is the case to some extent with epigenetic modifications of DNA, yet overall this is not what we see.

### Implications of the Hypothesis

One of the consequences of tree-thinking [63] is that genes are thought of as private goods or lineage goods (i.e. club goods in which the club strictly matches a monophyletic group). That is to say that if inheritance of genes proceeds in a tree-like fashion, with genes only passed down through time from ancestor to direct descendent, then genes are seen as being available only to one particular lineage, with other lineages being effectively excluded from using these genes (this would define genes as being excluded). The absence of reticulation in the tree of life hypothesis implies that, when genes evolve *de novo*, they become a synapomorphy for the descendents: only descendents of the entity in which the new gene first appeared will have the gene. This makes genes non-rival for this group of organisms and consequently genes can be throught of as club goods under a Tree of Life hypothesis. Animals for example, generally do not share their genes with animals of other species and more broadly with other organisms. That said, there is evidence that such events can and do occur. We see, for instance, genes from endosymbionts in the genomes of their hosts [64,65], multiple transfers of glycolytic enzymes into animals [66], transfer of the shikimate pathway into a basal metazoan [67] and genes enabling parasitism in nematodes [68] that are normally found in bacteria. Yet in general tree-thinking and the focus on the animal-based species concept, *sensu Mayr *[2], could lead us to view gene ownership to be a club where genes are kept for use only by members of the club. Hierarchical tree-thinking promotes an idea of a club within a club within a club. However, this situation, which is typical of animals is far from universal. Therefore tree-thinking cannot be suited for all biological evolution: goods thinking is an appealing alternative.

It is undeniable that tree-like patterns are also found by the analysis of phylogenetic trees from prokaryotes [23-26,31,69,70]. This is evidenced by gene trees constructed from samples of presumed orthologs that agree with each other far more than expected by random chance and that agree with each other far more than they disagree with each other [71]. This, however, should not be taken as evidence in favor of a general tree like evolutionary process and against the Public Goods hypothesis. According to the Public Goods hypothesis, we expect that if genes are public goods we would see a *local *tree-like pattern to emerge from the process of gene acquisition by a genome, followed by its inheritance by the offspring, however, we would also expect to see that there is no *global *tree-like pattern. This is exactly the pattern found by Schliep and co-workers [33]. In contrast, the Tree of Life hypothesis predicts a tree-like pattern throughout and this is not what we see [24,31]. We are careful to include Tree of Life concepts such as those envisioned by the "Statistical Tree of Life" or any of the other approaches that have fuzzy tree concepts. These hypotheses are 'regionalized' within the public goods hypothesis (we discuss regionalized hypotheses later).

Public goods genes are continuously acquired by genomes, they are integrated into these genomes and participate in transcription, translation and crucially, replication [72]. The replication of the genes and the cell in which the genes are found, facilitates the passage of these genes vertically from parent to offspring. Lineages in which the combination of genes is less fit than their competitors are removed and those lineages that produce more offspring that live long enough to reproduce in turn, are privileged. They form part of the selective landscape of the genome and population processes such as random genetic drift and natural selection act on these genomes, preserving those mutations that are advantageous or nearly so and rejecting those that are bad. Genomes do not have to use all the publicly available genes as this would lead to genome sizes that prokaroytes cannot support [73] and it would lead to a homogenization of all prokaryotic genomes so that they would all have the same genes. Clearly this does not happen. The ocean might be seen as a public good, but not everybody likes to swim, all the time.

When genes are seen as public goods, horizontal transfer of many gene families is possible. Not only is it possible, but it has almost certainly happened [39]. Dagan and co-workers have shown that in the history of life on the planet "at least two-thirds and probably all" genes have been affected by HGT at some time in their history [39]. The *rate of retention *of genes in a given genome is what is different. There is an obvious gradient of gene retention from high to low, with many genes that encode highly-connected proteins in the protein-protein interaction network being retained and co-evolving together for long periods of time [58], but other metabolic genes, antibiotic resistant genes, mobile genetic element genes, among others only being retained for very short periods of time (these timescales can range from one cell division to 4.5 billion years). The consequence of natural selection (*sensu *Darwin) retaining genes for varying amounts of time means that recent and ancient tree-like structures - some compatible, other not- might emerge, and this is precisely what we see [25,33].

**Figure 1:**
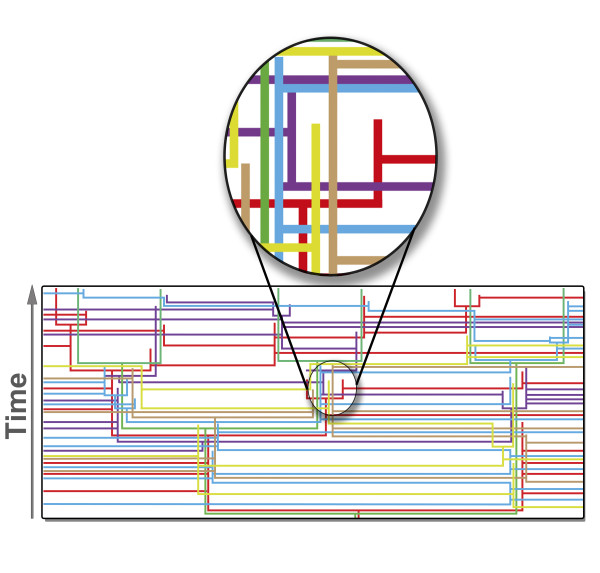
**This figure is a cartoon diagram representing the movement of genes from a variety of gene families as they are sampled by different genomes**. Each tree diagram embedded in this network represents the evolutionary history of that particular gene. Genes are continously sampled as public goods and this leads to anastomoses, coalitions and mosaic genomes. Part of this diagram is magnified to demonstrate that the local tree-like pattern that seems to emerge really just results from varying periods where genes co-exist in the same evolving entity.

Figure 1 shows clearly how local tree-like signals *must *emerge from a continuous process of sampling genes from the public/coalition pool of genes and their subsequent retention. Genes are acquired by a genome, retained for varying amounts of time, duplicated by DNA polymerases, (unless they produce an adverse or deleterious reaction in the organism), passed to offspring through cell division and are subsequently replaced by other genes, perhaps from the same or perhaps from different families, or they are simply lost.

One pattern that has been observed consistently across life is a pattern where genes naturally fall into two categories, delineated by the frequency with which they are involved in horizontal gene transfer. These are the "informational" and the "operational" classes of genes and the theory covering this observation is the "complexity hypothesis" [74,75]. According to this hypothesis, the complex interactions that information processing genes - those involved in transcription, translation and replication - are involved in makes it more difficult for these genes to be readily exchanged between organisms and so they tend to be inherited together more often than the operational genes which are involved in fewer interactions. In a very significant update to this hypothesis, Cohen *at al*., [58] have shown that the propensity for a gene to be horizontally transferred correlates negatively with the number of protein-protein interactions of its protein product. In fact, connectivity is much more important than function in determining the likelihood that a gene is involved in HGT. In other words, many more genes would become involved in frequent HGT were it not for their high levels of connectivity preventing the success of these events. The Tree of Life hypothesis does not predict that these kinds of pattern should emerge. However, according to the public goods hypothesis this is exactly the kind of pattern that is expected and indeed we view hypotheses like the complexity hypothesis to be regionalized within the public goods hypothesis.

Typically, we can describe the complexity hypothesis in terms of public goods as follows: sampling of genes from the public goods pool is a continuous process, however natural selection for fitter genomes in a constantly changing world decides whether these combinations of genes will work together for a short or long period of time. We therefore, expect to see variation in the rate of retention of genes. This creates a tree-like pattern, or a "central tendency" [31] for a tiny number of genes [25], while the majority of genes display a variety of other patterns with varying amounts of conflict to the 'nearly universal' genes [31,69] (and see also Leigh *et al. *[76] for an even finer description of the variation in the rate of retention of genes in the 'nearly universal' genes). We feel that such empirical observations from numerous studies fit the public goods hypothesis much more closely than any Tree of Life hypothesis.

### Regionalization of previous hypotheses

In 1854, Bernhard Riemann delivered a lecture at the University of Göttingen describing his theory that Euclidean space could be extended beyond the three or four dimensions that were being used at that time. This differential geometry of Riemann was subsequently developed and provided some of the foundations for Einstein's theory of relativity a half-century later. This development does not mean that euclidean geometry is not useful in some contexts, just that it is insufficient to explain all the data. Similarly, classical (Newtonian or Hamiltonian) mechanics, which describes the motion of bodies when force is applied, has given way to quantum mechanics, due to the fact that at atomic or subatomic scales, classical mechanics is unable to describe the observations of the behaviours of systems. Classical mechanics is still useful in a practical sense for macroscopic applications, however, it is insufficient to explain all mechanical interactions and indeed many quantum effects run counter to the expectation from classical mechanics [77]. In these examples, classical mechanics and euclidean space can be said to be regionalized instances within the universe of mechanics theories and geometrical spaces. In other words, these disciplines can be studied as long as the monist view is not taken that they are correct for all observations. We would advocate the pluralistic position that all Tree of Life and indeed many other, hypotheses are regionalized instances of the public goods hypothesis. The Tree of Life hypotheses can be studied and may have heuristic power (see Koonin and O'Malley, this special issue), but they are insufficient for describing all of biological evolution and indeed contradict many important observations.

### Summary

The public goods hypothesis for the evolution of life on the planet views some, perhaps most, genes as non-excludable, non-rival goods that are acquired by genomes for varying amounts of time before usually being replaced. From this continuous process of sampling of public goods, integration of the goods into a genome followed by genomic and cellular replication and division, we can see a net-like structure emerge with some local parts of this structure is expected to look tree-like (see Figure 1). The Public Goods hypothesis does not view any tree as being of particular significance as the process of evolution does not follow any particular tree and indeed tree-thinking is an incorrect starting point, likely to conflate explanandum and explanans. The Public Goods hypothesis is a much better fit to the observed data from genetic sequences, and it is not restricted solely to the evolution of cellular organisms, as it offers an important role and room for mobile genetic elements. It is consistent with the clear lateral connections between lineages that otherwise have little in common, it is consistent with the patchy distribution of most genes, it is consistent with the near-universality of the genetic code, it is consistent with the complexity hypothesis and it explains how we see a weak tree-like signal embedded in the net of life. It encourages predictions and testing of the extent of coalitions and barriers or 'road-bumps' to HGT in the environment (i.e. consistent with microbial ecology studies), and for the fundamental role of public goods in evolution, two features lacking from the Tree of Life hypothesis.

We view the complexity hypothesis [58,74], the selfish operon model [78], the Tree of Life model, endosymbiotic gene transfer [12], fusion models for eukaryogenesis [79,80], the arm races between systems favouring HGT and their corresponding defense mechanisms, even within mobile elements [56,81,82] and HGT hypotheses to be regionalized instances of the Public Goods hypothesis.

Finally, we feel that a fundamentally important feature of the public goods hypothesis is that it satisfies both of the axioms that we have outlined previously: axiom 1, that all evolving entities are included in the presumably highest-level evolutionary picture, and axiom 2 that genes can be inherited both vertically and horizontally. We know of no version of the Tree of Life hypothesis that satisfies axiom 1 and we believe that no version of the Tree of Life hypothesis sufficiently satisfies axiom 2.

We hope this manuscript, despite the relatively alien nature of the language in the evolutionary biology literature, provides an impetus for a programme of experimental and theoretical work testing the Public Goods hypothesis.

## Reviewers comments

**Reviewer 1: **W. Ford Doolittle (Dalhousie University, Halifax, Canada)

This paper is both fun and serious. There *is *a need to develop new ways of conceptualizing gene and genome evolution, globally, and borrowing from economics is potentially fruitful. I also agree with the authors' characterization of the state of play, that as a community we are now "straddling the middle ground - there is a great Tree of Life (*sensu *Darwin, Lamarck, etc) but it has annotations and complications superimposed upon its great frame, caused by interspecies gene transfer."

As well, I'd agree that "the Tree of Life hypothesis is almost unique in having been modified so extensively from its original description, in order to avoid its rejection". But I do wonder at the third author's acceptance of such a malleable notion, in the context of the 2007 paper [38] in which he and I were rather more specific about what we called the "Tree of Life hypothesis". We described it there as the notion that the tree-like patterns of "groups subordinate to groups" already recognized as natural before 1859 could be explained by (were caused by) an underlying tree-like pattern of successive lineage branchings. This is a testable casual hypothesis not least because there *are *alternatives, as presented by Peter Gogarten and colleagues elsewhere in this issue. And, we claimed, it was a cornerstone of Darwin's theory.

The chameleon-like Tree of Life hypothesis that seems to be referred to here (as a foil to the authors' Public Goods Hypothesis) must be a weaker conjecture, maybe something so vague as "There *is *a Tree of Life", or so vacuous as "Application of tree-building algorithms will build a tree", or (best case scenario) "There is a Tree of Cell Divisions that we can know, even if it is way less predictive than we had hoped". It is perhaps something like this, more casual than causal, that most tree-builders do have in mind and is thus referred to in this paper.

**Author's response:***We have been more explicit about Tree of Life hypotheses and indeed we are not talking about the casual ToL nor are we talking about the Public Goods hypothesis as something casual - we describe it as the basic model for producing the variation in heritable features that we see in evolving entities. We have included clearer statements on the ToL hypothesis such as "To put it another way, all formulations of the Tree of Life hypothesis have at their core the basic tenet that the pattern of diversity that we see on the planet is caused by a tree-like evolutionary process and the differences in these formulations is to be found in how much they allow deviation from this central idea"*.

I would quibble with the authors' assertion that the existence of CRISPRs and restriction-modification systems "testifies to the evolutionary success of (and obvious need for) mechanisms that protect against the (passive or aggressive) acquisition of genes". CRISPRs do seem to have evolved as protection against phages and maybe burdensome plasmids but it seem most unlikely that there is serious selection against importation of foreign genes *per se*, and restriction-modification systems may be selfish in nature [83].

**Author's response:***We agree with the comment on R-M systems being selfish in nature, but our comment on CRISPRs and R-M systems is simply the observation that these are genes that have evolved to counter the dangers of parasite DNA being incorporated into the host genome or cell. They can successfully prevent this from happening. This is in contrast to being eaten by parasites (antibiotic genes, for instance, function to resist being engulfed or outcompeted). Therefore, our difference in opinion might come down to the interpretation of the word "serious" in the phrase "serious selection against importation of foreign genes"*.

I also think there is much more that can be done with such public goods thinking in terms of what's in it for the genes themselves. If a particular sort of function is dispensable (what Lawrence and Roth [78] called a weakly selected function), and if the function can be performed by many different genes (and maybe even many different families of genes), there will be competition among such genes. How might one win out over others? Possibilities would be to have fewer prior co-evolved attachments (as in the "complexity hypothesis" of *Jain et al*. [75]) Another would to be not to use too many generally rare codons or expensive amino acids. A third might be to be short. It may be that such selected properties of public goods genes determine some of the global properties of bacteria and their genomes that we currently think of us selected at the level of cells and genomes, for instance the lesser level of connectivity (number of protein-protein interactions) of prokaryotic as opposed to eukaryotic proteomes. Once we start thinking this way it is not easy to stop.

**Author's response: ***We agree with all of this. "Goods thinking" will certainly provide a different perspective*.

**Reviewer 2: **Eugene Koonin (NCBI, NLM, NIH, United States)

This is an interesting and entertaining essay on "horizontal genomics" and the inadequacy of the Tree of Life hypothesis. To me, the most appealing part of the article is Table 1 and the accompanying text where the authors introduce Samuelson's classification of goods and advocate its use in phylogenomics for an evolutionary classification of genes. This classification is interesting and useful, and might even stick although I think the authors could have been more explicit in discussing it, for instance, whether some genes can and should be viewed as 'common goods' as opposed to 'public goods'.

**Author's response: ***We feel this is part of a multi-disciplinary programme of research and worthy of further publication at a later stage. For now, we wished to present the model*.

Without any pejorative implications, it is my impression that beyond this fresh interdisciplinary perspective, there is little in this manuscript that is genuinely novel. In more conventional terms, the "public goods" concept looks familiar. To honk my own horn for once, here is a quote from the abstract of a 2008 review article on genomics of Bacteria and Archaea: "...comparative genomics also shows that horizontal gene transfer (HGT) is a dominant force of prokaryotic evolution, along with the loss of genetic material resulting in genome contraction. A crucial component of the prokaryotic world is the mobilome, the enormous collection of viruses, plasmids and other selfish elements, which are in constant exchange with more stable chromosomes and serve as HGT vehicles. Thus, the prokaryotic genome space is a tightly connected, although compartmentalized, network, a novel notion that undermines the 'Tree of Life' model of evolution and requires a new conceptual framework and tools for the study of prokaryotic evolution" [84]. I believe the "public goods" view is right there, even though the language is different.

**Author's response: ***We have, of course, built on previous work and in particular, we have been very aware of the body of work that has emphasized the role of HGT, the problems with the ToL and the need for a 'new conceptual framework'. We feel we have cited many of the reviewers manuscripts and we naturally give as much credit as we can in the available space for others who have made these valuable contributions*.

I should further note that the authors could have been more specific about their understanding of the "Tree of Life Hypothesis". What is that hypothesis that they consider to be refuted by comparative genomics? Clearly, this cannot be Tree of Life sensu Darwin because in the 19^th ^century (and for much of the 20^th ^century as well), the very idea of evolving genes and genomes did not exist, and so the Tree of Life hypothesis could not have been properly explicated (the authors of this article do make a note to that effect). Neither is it the modern "Statistical Tree of Life" hypothesis (see our article with Maureen O'Malley in this thematic series [85]) because to refute that hypothesis, actual statistical analysis is required that would prove false previous conclusions on the detectability of a central trend in the "Forest of Life" [31]. It seems to me that the hypothesis McInerney *et al. *are so adamant about refuting is a straw man of "fully coherent evolution of (nearly) all genes in all genomes", with the implication that a 16S RNA tree or a tree of a small number of highly conserved genes could be an adequate Tree of Life. Surely, that hypothesis is blatantly false and hopeless, but it hardly has been ever formulated in those explicit terms. The original publications by Woese (e.g.,[6]) simply assumed that the Tree of Life was embodied in the 16S RNA phylogeny, without much conceptual analysis of what this assumption entailed. Conversely, in the genomic era, it has become clear early on that the Tree of Life concept needed serious revamping if it were to survive (e.g.,[14]). I think that the image of the straw man can be only implied by some publications defending the traditional Tree of Life even in the face of extensive HGT (e.g.,[25,86]). Certainly, these authors were well aware of the wide spread of HGT and simply have chosen to emphasize what they saw as the continued importance and heuristic power of trees of universal genes. That latter idea can be meaningfully debated even now but to that end, it would help to formulate more clearly the hypothesis for which a replacement is proposed.

**Author's response: ***We have been clearer in this revised version concerning ToL hypotheses - from the causal Tree as envisioned by Darwin and which is, we feel, still at the very heart of ToL hypotheses, to the modern searches for Trees among forests, which are more weakly causal, but nonetheless still focus on some weak tree-likeness. We have also addressed these ToL hypotheses in terms of an axiomatic approach*.

*In terms of 'refuting' the ToL hypotheses, we refute the causal notion of a Tree of Life because clearly it is false. We replace it with the causal idea that acquiring genetic goods, either vertically or horizontally, leads to the patterns we see today in the data and that it is not a "just so" story that can be represented by drawing a network, rather it is a causal story that says that genes (or portions of genes or collections of genes) are acquired in the same way that goods are acquired in the world of economics. We reject the causal notions of a ToL, not from the perspective of being incorrect analyses, but from the perspective of being inadequate as the highest-level hypothesis. To this end we have clarified our ideas about the regionalization of ToL hypotheses*.

**Reviewer 3: **John Dupré (University of Exeter, UK)

This paper nicely summarises the decline of the tree in microbiology, and provides for philosophers a rare but paradigmatic account of what Imre Lakatos [87] called a degenerating research programme. The "hard core"--the commitment to an underlying tree--is protected at all cost, despite ever-growing bodies of contrary evidence, by an ever more elaborate "protective belt". The next stage in the Lakatosian picture of scientific progress is the rise of an alternative and progressive research programme, and of course this is the project to which the present paper aims to contribute.

Somewhat tangentially to the question of the merits of theories old and new, I have quite frequently heard the suggestion that one of the most pressing needs in promoting some of the more radical ideas arising from contemporary biology is to find alternative metaphors as effective as those that have become so widely entrenched and disseminated, from the selfish gene and the struggle for existence, to the tree of life. The idea of (some) genes as public goods is an original and interesting contribution to this need. The interchange of concepts between biology and economics has been a productive one for two centuries, and this suggests an exciting new addition to this tradition.

**Author's response: ***Just to clarify - we see the public goods hypothesis as providing not only a metaphor (clearly the Tree of Life provided a diagram/metaphor that could be used to describe evolving cellular life), but we see the Public Goods hypothesis as a description of the process of evolution - genes as goods that can be utilized, exchanged, protected, etc. So, while we would say that this is a metaphor, it is also a very real description of how genes are used by cells, viruses, plasmids etc. Goods thinking implies process as well as pattern*.

### Second Review by John Dupré

I did not mean to imply that metaphors were suspect tools in science; quite the opposite: they are crucial to scientific practice. In this regard, I agree with the authors about the importance of finding the appropriate concepts for evolutionary analysis.

### First review by John Dupré

There are, of course, a few questions that will need to be pursued further before this metaphor becomes widely accepted. Most obviously, and fundamental to the whole debate from which the suggestion arises, is the extent to which genes are really non-excludable goods. I take it that all genes are non-rival, unless perhaps for some very special situations involving competition within a species. Tree aficionados, then, will take all genes to be club goods, excluded to all except for a club often containing no more than the members of a species. Although different clubs may, by reason of homology, have access to the same gene, there is no trading between clubs and eventually their products will presumably drift apart. It is worth noting that the clubs may be a lot bigger than has often been supposed. Contrary to a strict interpretation of McInerney *et al*.'s remark that 'animals... do not generally share their genes with animals of other species' it now appears that there is a great deal more hybridisation than was once supposed [88]. But while this may produce larger clubs and perhaps even clubs with fuzzy membership rules, this is a long way from a fully non-excludable public good.

**Author's response: ***We call this hypothesis the "Public Goods" hypothesis to emphasise that genes, by virtue of using the same four (five) nucleotides, by using triplets of nucleotides in codons, by widespread use of the exact same genetic code and by virtue of known mechanisms of gene transfer, have the theoretical property of being public goods, but might find themselves in reality being part of a club (because of variant genetic code, function of the encoded protein, etc.). Goods are generally found somewhere on a gradient between one extreme of description and another, the categories are not discrete. However, there is indeed a programme of research in trying to understand the extent to which genes can be viewed, in reality, as public, private, club or common goods*.

The important question to begin with, anyhow, is whether the public good concept is useful for understanding the evolution of prokaryotes, and the extent to which it remains so for various parts of the eukaryote, or perhaps just the sexual, world can wait. Two billion years of cellular evolution would make a good start. I take it that economists tend to think of a public good as something easily available to anyone. An interpretation of this for the evolutionary case might be, a public good gene is one such that any lineage for which it would be useful, i.e. that would be fitter with the addition of this genetic resource, will find it. I leave microbiologists to say how close reality comes to this, but I suppose that it would be unusual at best to achieve this fully. As McInerney *et al. *say, there is a good deal of variation in the ease with which different genes can be horizontally transferred, and there is also variation in the competence of different lineages to acquire genes. It might be in the end that a more literal-minded exploration of this metaphor would lead to a quite complex mapping of the various channels and obstacles to genetic trade.

Central to the variation just mentioned between degrees of excludability of genes, McInerney *et al. *suggest a dichotomy between informational and operational genes, and it is operational genes, due to the greater simplicity of their embedding in the functioning of the organism, that are liable to be public goods. Here the analogy with economics begins to limp. In the economic context information is a key example of a public good, perhaps in the age of the internet it has become the most important example of a potentially public good. It is the informational character of genes that underlies the non-rival character central to the possibility of their being public goods. Perhaps one way out of this rather confusing situation would be to replace the dichotomy just mentioned with one between information-processing genes as opposed to mere informational genes (i.e. operational genes). Alternatively, as a general sceptic about dichotomies, I am more tempted to suspect that this is more of a continuum of embeddedness and transferability, and as such might fit well enough into the mapping suggested at the end of the last paragraph.

**Author's response: ***While this manuscript has been written, a very important manuscript *[58]* has emerged that has updated the complexity hypothesis and we have written about this in the revised version of the paper*.

A final reflection on the public goods analogy is the following. Perhaps the most fundamental issue involving public goods in economics is the possibility of free riders to which it gives rise; and of course the possibility of free riding has figured in many evolutionary discussions. It would be a strong endorsement of the public goods metaphor if parallel issues could be discovered in prokaryote evolution. In some sense a cell or a cell lineage that avoids the cost of carrying a gene (say antibiotic resistance) that is not currently needed, safe in the 'knowledge' that some other lineages were keeping it readily available, might be said to free-ride. Is there a story to be told about the division of the costs of maintaining the stock of genetic resources in an otherwise free genetic commons? If so, the metaphor might show itself productive as well as expository.

**Author's response: ***Indeed free riders do exist - plasmids that are non-conjugative can only transfer with the assistance of conjugative plasmids and so these are candidates for the category of 'free-riders', though this is only a suggestion. As we said previously, the current manuscript is designed to propose the Public Goods hypothesis and to propose "goods thinking" instead of "tree-thinking". Future work will be needed to evaluate free-riders, clubs, genetic commons, etc*.

In summary, I am not yet convinced that the public goods hypothesis is a metaphor with the potential to do all the theoretical and public relations work formerly carried out by the Tree of Life. But it is at worst an intriguing way of raising a range of interesting questions, and may yet develop into a powerful and productive intellectual tool.

**Reviewer 4: **Gregory Morgan (College of Arts and Letters, Stevens Institute of Texhnology, United States)

Over the last two centuries, there have been many instances of the lateral exchange of ideas between biology and economics. Famously, Darwin was inspired by Malthus's work on reasons for declining living conditions in 18^th ^century England [89,90]. Social Darwinists like Herbert Spencer thought that biological concepts like "survival of the fittest" also applied in economic contexts [91]. More recently, game theory developed outside of biology has been used to analyze evolutionary stable strategies [92].

McInerney *et al. *propose another exchange: that biologists should co-opt the economic concept of public goods for biology and think of genes or sets of homologous genes as public goods. They cite the economist Paul Samuelson as the originator of the idea of public goods, although there are earlier versions of the concept going back as far as John Locke's discussion of property [41].

**Author's response: ***We have now made this comment in the main body of the manuscript*.

If the Public Goods Hypothesis is advanced as a metaphor, then it might supplement the tree of life. Presumably, McInerney *et al. *do not treat the Public Goods Hypothesis as a mere metaphor since they claim the Public Goods Hypothesis is an alternative to and indeed a replacement of the Tree of Life Hypothesis. What is literally true of (many) genes, they contend, is that they share with public goods the properties of being non-rival and non-excludible.

**Author's response: ***This is correct, we do not see the Public Goods hypothesis as providing an alternative as a wooly metaphor, we feel that it is a sensible way of looking at how genes are treated by evolving entities - as goods that might or might not be used in different circumstances and as such, these genes have properties that are also recognized in things like cups of coffee and air*.

What these concepts mean in a biological context is not made completely clear. By non-excludible, they say, "it is impossible or a least very difficult to exclude the good from being available to everybody." In the biological case, presumably one replaces "everybody" with "every genome" or perhaps "every genotype over a certain amount of time." Depending on what time scale we select, a gene might vary in its excludability. Perhaps McInerney *et al. *could quantify "very difficult" with a certain probability of horizontal exchange in a given unit of time to make what counts as a public good in biology more precise.

**Author's response: ***We have cited the very important paper by Cohen et al. where they quantify the rates of HGT across a large sample of genes and show how it correlates with the connectivity of the encoded protein product. However, the rate of HGT and excludability may be two different things, though this needs further investigation. We still have outstanding questions such as whether or not all the gene sequences within, say, a particular ribosomal protein family are so well adapted to the ribosome in which they find themselves that they are effectively excluded from ever becoming involved in an HGT event. Given the limits of what can be done in any one manuscript, we feel that this is a significant question that warrants investigation, but is outside the scope of the current manuscript*.

Unfortunately, a gene's probability of transfer will vary depending on the nature of the recipient genome in question. The concept of being "non-rival," too, needs further elaboration in the biological context. The idea is that a gene can be laterally transferred into a different organism without being "used up." But what does it mean to say that a copy of a gene on one organism precludes its use in another organism? One way to examine this question would be to look at what effect transfers of the gene has on the fitness of its instances. If an increase in copy number of a gene in a population decreases the fitness of any given instance of the gene, then there is a sense that its adaptive utility is being "used up" and the various copies are to some extent rivals with one another. On this suggestion, being a rival is a matter of degree. At one extreme when the utility of a gene is "all used up" because of prolific copying there is now selection against the gene in the donor genome in question. Perhaps there are other ways also to elaborate what it means for a gene to be "used up," but the authors do not pursue them. In any case, as one attempts to make the Public Goods Hypothesis more precise, the hypothesis becomes more complicated.

**Author's response: ***Goods are generally viewed as being somewhere along the gradient from one extreme to the other extreme and the neat classification of goods is often not possible in reality. We gave the example of clean air, which is normally a public good, but for deep-sea divers with bottled air, it becomes a private good available only to the owner of the tank of air. In terms of rival genes, if a gene becomes too common in a particular environment it is likely that there is overuse of the environment and therefore, genes can become rival. Again, we feel that this is part of a progressive research programme to evaluate these kinds of genes*.

One virtue of the Tree of Life Hypothesis is its simplicity, especially before the use of reticulation to account for lateral gene transfer. It was applied to all of life and any organism could in theory be placed at one and only one place in the tree. The Public Goods Hypothesis is not as simple. While many (McInerney *et al. *say "perhaps most") genes can be thought of as public goods, others cannot. Some should be thought of as being "club goods" i.e., they are excludible but non-rival. Furthermore, as McInerney *et al. *point out, what type of good a gene is classified under will depend on context. The tree of life offers powerful visual images that summarize patterns of relatedness. Is it possible for the public goods hypothesis also to offer an efficient and powerful means of visualizing patterns of relatedness? It is not clear.

**Author's response: ***First of all, we would contend that the Tree of Life hypothesis was indeed applied to all of organismal life, but not to all evolving entities - it conspicuously left out plasmids, viruses, phage etc. Simpler ideas are appealing and indeed parsimonious reasoning is a powerful argument that is applied for many explanations in biology. However, evolution is dynamic and in our opinion a dynamic, pluralistic interpretation of the evolution of life on the planet is needed. The elegance, or otherwise, of the model is subjective*.

On a positive note, if McInerney *et al. *are correct in claiming that the public goods hypothesis predicts local tree-like structure and global non-tree-like patterns, then this is a significant point in favor of the hypothesis. To make this point more strongly, a quantitative dynamic model of public goods that shows how patterns like their Figure 1 might be produced would be of interest.

**Author's response: ***We agree with this, but feel that quantitative analysis is best left for another manuscript. In this current paper, we are explaining the model*.

To make the public good hypothesis more plausible and move it beyond a speculative hypothesis, it would be useful to quantify the evidence in its favor. McInerney *et al. *claim that their hypothesis describes the genomic data better than the tree of life, and they may be right, but they do not offer any quantitative measures of degree of fit. In the longer run, proponents the Public Goods Hypothesis need testable novel predictions that cannot be explained, or at least explained only poorly, with a tree, sets of trees, or trees with reticulated branches. In sum, the Public Goods Hypothesis is a cute idea, but needs further quantitative development before it rises to the level of a genuine challenger to the Tree of Life Hypothesis.

**Reviewer 5: **Davide Vecchi (Universidad de Santiago de Chile, Chile)

The idea of the authors is to use concepts originally formulated in economics in order to offer a categorisation of genetic resources. This interdisciplinary ethos is to be welcomed, if only because contemporary science is too compartmentalised. The big question is whether the ensuing categorisation is effectively carving nature in an interesting and heuristically useful way.

### Heuristic power

The authors contend that the analogy of genes to public goods and club goods is at odds with the Tree of Life (ToL) hypothesis. They also argue that the idea that genetic resources can be categorised using the concepts of rival and excludable goods makes sense of various observations (e.g. that it seems difficult for prokaryotes to prevent other prokaryotes from eventually exploiting a particular genetic resource). There is no doubt that using such new categorisation is in principle heuristically useful. In fact, it could highlight phenomena that possibly were previously considered of secondary interest. For instance, consider the existence of genetic *clubs*: the public goods (PG) hypothesis emphasises the theoretical significance of questions such as "why do such clubs exist?" and highlights the importance of studying the phenomenon of genetic retention within clubs. Using different theoretical tools and analogies - even drawn from distantly related sciences - with the aim of understanding in novel ways biological reality is highly commendable.

Nevertheless, even though the PG hypothesis could generate interesting insights, many questions remain concerning its theoretical status. One is whether the PG hypothesis is on a better epistemic footing than the ToL hypothesis, as argued by the authors. Another is whether it is general enough to be applicable to all genetic resources. A third is whether it is general enough to be applicable to all kinds of shared resources. I will comment on these issues in turn while also asking unashamedly speculative questions.

### Saving the phenomena

The authors claim that the ToL hypothesis is almost unique in having been modified so extensively from its original proposal. This seems to me an overstatement. An interesting aspect of the history of geocentrism concerns the creativity of astronomers and the variety of solutions they devised in order to save the observed phenomena. This process lasted nearly twenty centuries. Is the ubiquitous practice of devising "epicycles" (aka ad-hoc hypotheses) justifiable? Many philosophers of science have argued that it is not. As a matter of fact, it looks more like a functional element of science: defending one's hypothesis is necessary for the dialectical process of theory change. This could also mean that there is no expiry date for the ToL hypothesis, even though all that is left of the original proposal is a rather unimpressive "tree of 1%*"*.

**Author's response: ***We 'toned down' this sentence about the ToL being 'unique'. We recognize that it is not unique in being stretched so far from its original proposal. We have also been careful to say that the ToL if it is properly interpreted (though there are so many interpretations it is difficult to keep track) can be considered a regionalized hypothesis within the public goods hypothesis*.

The problem always concerns the quality of the alternative to the mainstream hypothesis. When the authors sustain that the PG is the *best explanation *of the evidence of lateral connections between lineages, critics are bound to be sceptical, mainly because the PG hypothesis is designed in order to *accommodate *these data. The critical question is whether the PG hypothesis can bring something new to the fore (e.g. unexpected and successful predictions concerning novel kinds of phenomena). Can it?

**Author's response: ***When we formulated this hypothesis in 2010 we proposed, privately, that there are more mechanisms of gene transfer that have yet to be discovered and then in 2011 there has been the publication of nanotubes being observed ferrying genes fro one cell to another *[43]. *We feel that goods thinking has many predictions concerning gene transfer, defence against transfer and so on*.

### Second review by Davide Vecchi

The authors clarify in what sense the PG hypothesis is a radical alternative to the tree of life hypothesis. They say that the essence of tree thinking is that evolution is a process of continuous divergence. Tree thinking is inconsistent with the existence of HGT and of the mobilome. The alternative is that evolution is a continuous process of sampling of genes from the public goods pool. From this perspective the tree of life hypothesis is the incorrect starting phylogenetic assumption, the wrong null or default hypothesis, the reason being that the "primitive evolutionary state" is the public state. Seeing evolution as a process of continuous divergence and seeing evolution as a process of sampling from a public pool of goods are two radically different perspectives.

**Author's response: ***This is indeed what we have been trying to achieve for this manuscript. We feel you have summarized it very nicely*.

### Davide Vecchi continues

The epistemic advantages of the PG hypothesis are many: it accommodates the evidence in favour of the existence of extensive HGT; it "makes unsurprising" (i.e. explains) the inevitable existence of the mobilome (given the nature of the ancestral evolutionary state); it makes sense of the existence of many evolving entities that do not belong to the cellular level of biological organisation. In addition to such accommodating roles, however, the PG hypothesis also fulfils other epistemic roles: it focuses its research on the discovery of new mechanisms of gene transfer [43] as well as on the discovery of barriers to HGT that have evolved in the history of life; it encourages testing of the extent to which coalitions of genes or club goods affect evolutionary dynamics; it provides the theoretical basis for new experimental results and new predictions (such as the role of genomic connectivity [58]) etc. In all these senses the PG hypothesis is clearly more than a metaphor.

**Author's response: ***Yes, we have been trying to infer that it is more than a metaphor - it describes something causal*.

### Unification

The aim of the ToL hypothesis is to unify: the pattern of evolution is assumed to be the same for all organisms, multi-cellular eukaryotes and prokaryotes alike. Nonetheless, it turns out that the ToL creates a big divide: there is a fracture between *normal *organisms (those for which trees can be constructed) and "freaks" (for which we cannot). The PG hypothesis attempts to provide a picture of biological evolution that does not treat microbial phylogenetics as a special case, and that tries to finally re-unify microbial freaks and eukaryotes. But I wonder whether the PG hypothesis runs the risk of creating a converse divide, one where eukaryotes assume the new role of freaks of nature. The PG hypothesis rests on the implicit assumption that it will eventually be discovered that the same interactive and cooperative dynamics that affect the world of microbes also affect the world of macrobes. However, what are the theoretical reasons behind this implicit assumption? Why should we consider the PG hypothesis as general enough to encompass all genetic resources, both prokaryotic and eukaryotic, given the different systems of inheritance? Finally, in the eventuality that micro and macrocosms turn out to be governed by deeply different processes, what will be the consequences for the old idea of biology as a unified science of *all *life?

**Author's response: ***Obviously, the use of the word 'freaks' entails a particular perspective that may not be held by all. Multicellular organisms engage in hybridization, we have seen Wolbachia genomes with perfect pieces of their hosts DNA integrated, we see transposons in multicellular organisms, so for sure nobody is completely excluded from accessing goods - vertical descent ensures that goods are available. Although the ocean might be considered to be a pubic good, we don't all have to go for a swim. There are many theories for why multicellular eukaryotes engage less in HGT than single cell prokaryotes, but labeling one or the other as freaks seems unnecessary*.

### Second review by Davide Vecchi

I think the new axiomatic approach of the paper is problematic. In the first place, the two axioms presented by the authors strike me as not being uncontroversial. The authors call them in turns "axioms", "conditions" and "desiderata", showing that there might be some confusion about their ontological status. Clearly, axiom 2 was a recent empirical discovery, while axiom 1 has even deeper problems because, as it is formulated, is eminently ambiguous: what are the "evolving entities"? Why should we take all of them into consideration? And what does "highest level evolutionary picture" mean?

These are difficult questions.

**Author's response: ***We have endeavoured to clear up some of these issues by stating that we mean all nucleotides that are to be found in a replicating system. The fact that axiom 2 is a consequence of recent scientific findings and not older findings does not negate its usefulness as a self-evident fact now*.

### Davide Vecchi continues

That the axioms are not self-evident can be shown very simply. It is sufficient to take Mayr's position into consideration. In a famous exchange with Woese, Mayr [93] proposed the following argument: evolution is about phenotypes; prokaryotes show uninteresting phenotypes; hence evolution is about eukaryotic phenotypes. Here we have, clearly, a biased conception of prokaryotes as "freaks" as well as an equally biased (i.e. non pluralistic) interpretation of axiom 1. The point is that, according to Mayr, the only evolutionarily important entities are phenotypes. Additionally, clearly not all kinds of evolving entities can be taken into account in order to build an informative evolutionary picture. For these reasons my opinion is that Mayr would deny the legitimacy of axiom 1, as would many other taxonomists. More generally, axiom 1 does not propose a solution to the problem of establishing what the units of evolution are. It is subject to interpretation, and until the interpretation favoured by the authors is not universally accepted, there will be division between accounts of prokaryotic and eukaryotic evolution. And there will be freaks.

**Author's response: ***Our interpretation of Mayr's ideas are somewhat different. Mayr said that "Evolution is an affair of phenotypes. It is phenotypes, not genes, that are the objects (targets) of selection" *[93]. *Mayr, in advocating that there were just two fundamental kinds of organisms simply said that the phenotypic similarities of eubacteria and archaebacteria logically placed them into one category and the phenotypic diversity of the eukaryotes identified them as a different category. In contrast to what is being asserted here, Mayr said in the same manuscript "[...] the possession of certain ancestral characters is often the most characteristic feature of a taxon." Therefore, we do not believe that Mayr found archaebacterial and eubacterial phenotypes uninteresting - in fact he found their relative lack of phenotypic diversity to be very interesting indeed. So, we will have to differ on this issue and re-state that we feel that both axioms are indeed uncontroversial*.

### Sharing resources

My last comment concerns the way in which communal goods are conceptualised by the authors. They only refer, understandably, to genetic goods, without considering the eventuality that a variety of gene products or other higher-level (e.g. phenotypic) resources could be exchange material in the microcosm. In fact, DNA resources are not the only types of resources that can be transmitted and shared. Contemporary theoretical biology also emphasises ecological inheritance, namely the inheritance of the set of environmental resources that are needed for the reconstruction of the phenotype. Can the PG hypothesis be enlarged as to encompass all kinds of resources (i.e. genetic, phenotypic, ecological and environmental)?

**Author's response: ***We feel that the Public Goods hypothesis emphasizes ecology much more than tree-thinking does, however we are careful to say that this current manuscript really does focus on the genetic elements and not on proteins, which have long been viewed through the lens of goods-thinking*.

I am asking this because I believe that even cultural change can be partially understood by means of the conceptual tools introduced in this paper. In fact, in cultural change cross-lineage borrowing mediated by mobile elements is very common and some cultural items are clearly public goods. However, the big difference is that in culture shared resources and mobile elements are not solely genetic.

Put another way, the primacy of genes as the sole resource of evolutionary interest is criticised in many parts of "eukaryotic biology" (e.g. developmental biology). The same idea requires profound extensions in order to make sense of cultural evolution. Nonetheless, genetic resources seem to be the sole focus in this paper, and possibly in microbiology. I wonder what the authors think about the prospects of unifying the study of evolutionary processes under a framework that takes into account all kinds of material resources exchanged and shared between organisms and lineages.

**Author's response: ***We have tried in this most recent draft of the manuscript to emphasise that genes do not exist in isolation from their genetic code, the functions of the proteins they produce or the content of the rest of the genome in which they find themselves. We are not sure if there is a unifying theory of all goods thinking, but it is certainly an avenue worth pursuing*.

## Competing interests

The authors declare that they have no competing interests.

## Authors' contributions

JMcI conceived the work and drafted the first version of the manuscript. DP, EB and MOC contributed ideas, corrections and substantial amounts of text to subsequent drafts. All authors read and approved the final manuscript.
